# Supplementary material for: Targeting RNA structure in SMN2 reverses spinal muscular atrophy molecular phenotypes
Source: Nat Commun. 2018 May 23;9:2032. doi: 10.1038/s41467-018-04110-1 (PMC5966403; doi:10.1038/s41467-018-04110-1)
Supplement: Supplementary file 1 — Supplementary Information [file 41467_2018_4110_MOESM1_ESM.pdf]

Supplementary Information

Targeting RNA structure in *SMN2* reverses Spinal Muscular Atrophy molecular phenotypes

Amparo Garcia-Lopez, Francesca Tessaro, Hendrik R.A. Jonker , Anna Wacker , Christian Richter, Arnaud Comte, Nikolaos Berntenis, Roland Schmucki, Klas Hatje, Olivier Petermann, Gianpaolo Chiriano, Remo Perozzo, Daniel Sciarra, Piotr T. Konieczny, Ignacio Faustino, Guy Fournet, Modesto Orozco, Ruben Artero, Friedrich Metzger, Martin Ebeling, Peter Goekjian, Benoît Joseph, Harald Schwalbe & Leonardo Scapozza

Supplementary Methods

Nuclear Magnetic Resonance (NMR) experiments

Sample preparation. Unlabelled RNA oligomers were chemically synthesized by GE Dharmacon and delivered in lyophilized, deprotected form. Selectively ^{13}C-^{15}N-adenine-labelled oligos were synthesized by Dharmacon, with isotope-labelled adenosine-phosphoramidites from Cambridge Isotope Laboratories, Inc. (CNLM-3806-CA-0). The lyophilized RNA was dissolved in NMR-buffer (10 mM sodium phosphate buffer pH 6.4, 50 mM NaCl and 0.1 mM EDTA). NMR spectra were measured either in 10 % D_2O/90 % H_2O or 99.8 % D_2O in DEPC-treated 5 mm shigemi tubes. For ligand binding experiments, 40-50 µM unlabeled 19mer was measured in NMR buffer containing 10 % DMSO and 7 % D_2O. Pre-heating of the sample to 90 °C for 5 min and fast cooling on ice resulted in >90% monomeric conformation. The 19mer RNA tends to self-associate at concentrations around 1 mM and in the presence of Mg^{2+}. Therefore, NMR experiments were carried out using diluted samples of max 0.6 mM in the presence of EDTA and absence of Mg^{2+}. Native gel electrophoresis and reproducibility of 1D imino proton spectra[1] were used to confirm sample integrity.

Chemical shift assignments. All assigned resonances are deposited in the BMRB (ID: 34100). ^{1}H-^{1}H NOESY spectra[1] and natural abundance ^{1}H-^{15}N-correlation spectra[2] in 90 % H_2O at temperatures from 5 °C – 25 °C were used for identification and sequential assignment of exchangeable imino and amino protons. ^{1}H-^{1}H NOESY[3,4] and TOCSY spectra[5], and natural abundance ^{1}H-^{13}C-correlation spectra[6] in 99.8 % D_2O at temperatures from 5 °C - 25 °C were used for identification and sequential assignment of non-exchangeable protons. Sequential assignment exploits the NOE connectivities between anomeric H1'-protons and the aromatic proton of the 3'-residue as well as the NOE between adenine H2 protons and the anomeric proton in the 3'-direction of the own strand and of the opposite strand in A-helical RNA. Ambiguities during the assignment procedure could be overcome by the use of an RNA sample with ^{13}C,^{15}N-labelled adenines. By ^{12}C-filtered (t_1+t_2) NOESY, ^{12}C-filtered (t_1) NOESY][7-9], ^{13}C-edited (t_1) NOESY[10], and 3D HCCH-TOCSY[11] experiments, all adenosine protons and 84 % of the total non-exchangeable protons were assigned. Furthermore,

employing 2D constant-time ^{13}C-^{1}H-correlation spectra[12,13], all adenosine carbons and 72 % of the C, U and G ribose carbons were assigned.

Secondary structure prediction as well as several chemical probing experiments suggest that the 19mer sequence forms an A-form double helix capped by a triloop consisting of residues 5'-$A_9A_{10}U_{11}$-3', with some evidence that the loop-closing base-pair is unstable, resulting in temporary pentaloop conformation. ^{15}N-HSQC spectra and ^{1}H-^{1}H-NOESY spectra of exchangeable protons at different temperatures confirmed that above 5 °C, only base pairs C4G16 to U7A13 are stable, whereas the imino protons of U3, U2 and G18 show strong cross signals to water, implying rapid exchange. 1D imino proton spectra show the U3 imino signal and broad resonances for G18 and U2 only at 5 °C. The imino proton of the loop-closing base pair, U12, is not detectable at any temperature tested. However, in the "sequential walk" of anomeric-to-aromatic protons, no interruption occurs until residue A9, strongly hinting at A-helical stacking for all base-pairs from A1-U19 to A8-U12.

Dihedral constraints. The shape of the ribose sugar puckers were determined semi-quantitatively based on the canonical coordinates calculated from ^{13}C chemical shifts for ^{13}C labelled adenine residues[14,15] and on TOCSY cross peak intensities between H1' and H2' protons for all residues, which are not detectable at short mixing times for 3'-endo puckered ribose rings and have medium intensity for C2'-endo ribose. Glycosidic torsion angles were categorized according to the intra-residual NOE cross peak intensity between the anomeric and the aromatic proton, with none or very weak NOEs corresponding to the anti-conformation common for A-form RNA and medium to strong NOEs corresponding to syn-conformation. A summary of all experimental constraints (distances) is provided in Table 1.

Restraints. Due to the absence of ^{15}N-labelled imino groups, hydrogen bonds could not be directly detected. However, given the exchange-protected imino protons of residues U6, U7, G15 and G16 at all temperatures and of residues U3, U2 and G18 al low temperatures, typical values for Watson-Crick base pairing were assumed. Base pair planarity was restrained for the base pairs U3A17 to U7A13 and, more loosely, for U2G18 and A8U12. Backbone torsion angles (α-ζ) were loosely

restrained for the A-form RNA stem, (base pairs U3A17 to U7A13) with δ corresponding to v_3 in the ribose ring. A summary of all restraints is provided in Table 1.

Structure calculations. The NMR structure calculations were perfomed with CNS 1.1[16] using the ARIA 1.2[17] adapted setup and protocols with the nucleic acid forcefield including OPLS charges and nonbonded parameters[18]. The standard annealing protocols were used, including NOE distance calibration (separated for the water-exchangeable protons) and spin-diffusion correction. Dihedral angle restraints, Watson-Crick basepair hydrogen bonds and planarity restraints as described above and in Table 1 were included. The chemical shift resonances were manually assigned and the 2D ^1H,^1H-NOESY spectra were manually peak picked and assigned to a large extent using Sparky[19]. The resonances and 9 NOESY peak lists (from two 2D ^{12}C-filtered NOESY spectra, 900 MHz, 200 ms mixing time; a ^{13}C-editted NOESY spectrum, 900 MHz, 100 ms mixing time; 2D homonuclear NOESY spectra either in D_2O, 900 MHz, 100, 200 and 300 ms mixing time; or H_2O, 900 MHz, 300 ms mixing time and 600 MHz, 50 ms mixing time; and a 3D NOESY-^{13}C-HSQC spectrum, 800 MHz, 150 ms mixing time) were used as input for automated cross peak assignment and calibration with ARIA. The proton chemical shift tolerance was set to 0.015 ppm in both proton dimensions. 100 starting structures were generated based on a linear template molecule. For each iteration (0-7) in which 100 structures were calculated, the NOE distance restraints were recalibrated by ARIA based on the 20 lowest energy structures. The violation tolerance was progressively reduced to 0.1 Å in the last iteration (8) in which 400 structures were calculated. For the structure calculations, a four stage simulated annealing (SA) protocol was used using torsion angle dynamics (TAD). The high temperature stage consisted of 10000 steps at 10000 K. This was followed by refinement and cooling down stages: 8000 steps at 2000 K, 20000 steps to 1000 K and 15000 steps to 50 K. During the SA protocol the force constant for the NOE restraints was set to 0, 10, 10 and 50 kcal mol^{-1} $Å^{-2}$ for the successive stages. The final 40 lowest energy structures were further refined in explicit water[20] and have been deposited to the PDB (5N5C) and BMRB (34100). All input scripts used are available upon request.

Ligand titration experiments. The ligand PK4C9 is poorly soluble in water, therefore the titration experiments were carried out in 10 % DMSO at low RNA concentrations (40-50 µM). Up to 20

equivalents of ligand were added from a 100 mM solution in 100% DMSO and changes upon ligand titration were monitored for the imino proton pattern by jump-return echo 1D spectra, for the pyrimidine H5/H6 proton pattern by TOCSY spectra with short mixing times[21], and for the adenine aromatic and anomeric protons by ^{13}C-HSQC spectra[12,22,23]. Chemical shifts induced by PK4C9 were only observed in TOCSY spectra showing the cross peak region of H5/H6 protons of the pyrimidine bases. ^{1}H chemical shifts were referenced to the internal DMSO signal at 2.63 ppm. The residues closest to the helix end, U2 and U19 showed the most significant shifts, clearly distinguishable from the DMSO-induced shifts.

RNA sequencing

Four total-RNA extractions of DMSO-treated GM03813C fibroblasts (control) and four extractions of PK4C9-treated (40 μM, 24 h) GM03813C fibroblasts were obtained using the RNeasy mini kit with on column DNase digestion (Qiagen). RNA concentration was quantified in a Qubit fluorometer and integrity assessed with Agilent's 2100 bioanalyzer using pico chips. Samples with RIN values ≥9.5 were sent to Beckman Coulter Genomics for RNA sequencing. A poly-A capture protocol was applied to eliminate tRNAs and rRNAs prior to cDNA synthesis. Eight Illumina TruSEQ RNA-seq libraries were constructed and sequencing was perfomed on the Illumina HiSeq 2500 platform using paired-end 125 bp read lengths. A total of >400M read pairs were produced, of which >90% could be mapped to the human genome reference sequence GRCh38.

RNA-seq raw data processing. To estimate gene expression levels, paired-end RNA-seq reads were mapped onto the human genome (hg19) by using the short read aligner GSNAP[24,25] and RefSeq transcript annotations. Mapped reads for all transcript variants of a gene (counts) were combined into a single value, normalized and denoted as rpkms (number of mapped reads per kilobase transcript per million sequenced reads)[26].

For the splicing analysis, paired-end RNA-seq reads were mapped onto RefSeq human transcripts using the alignment software GSNAP with default parameters and the option 'sam-multiple-primaries' in order to account for reads that map multiple times to different splice variants. The number of reads spanning splice-sites was determined by applying the samtools mpileup software[27]

with default parameters for each transcript. Reads were excluded from the analysis if they were not mapped as proper read pair or their start or end coordinates mapped exactly onto the splice-site. RNA-seq data have been deposited in Gene Expression Ommibus (GEO accession number GSE94111).

Alternative splicing analysis. The sequencing reads generated do not allow unambiguous identification of full-length transcript variants. Therefore, a database of local alternative splicing events was generated based on the human RefSeq database of transcripts (Release 66, August 2014). Such local splicing events were classified into the following categories:

CASSETTE – inclusion or skipping of a single exon

INTERNAL3 – use of an alternative 3' acceptor splice site, leading to a length polymorphism

INTERNAL5 - use of an alternative 5' donor splice site, leading to a length polymorphism

LCASSETTE – joint inclusion or skipping of two exons

MUT_EXCLU – mutually exclusive usage of one out of two exons

XCASSETTE - joint inclusion or skipping of three or more exons

For each local splicing event, a distinction was made between two alternatives, e.g. including or skipping the exon in the case of a CASSETTE event, and all the sequencing reads that were unique for either alternative were collected; for the CASSETTE case, these were reads covering the different splice junctions or mapping fully to the alternative exon. The obtained read counts for either alternative were normalized by the length of their corresponding unique sequence stretches (which is typically longer for the exon inclusion alternative) to obtain normalized counts c1 and c2 for the two alternatives, respectively. Splicing events were then characterized by a PSI ("percent spliced in") score PSI = c1/(c1+c2), which ranges from 0 to 1. Changes were determined by ΔPSI values (relative to matched controls).

Additionally, exon junctions with read support only in the treated but not in the control samples were analyzed. Sequencing reads were aligned with STAR (version STAR_2.5.2a) and GSNAP (version 2017-05-08) onto human genome hg19 by using default mapping parameters. SAM to BAM file conversion and PCR duplicate removal was performed with SAMTOOLS (version 1.5). By using the NH tag in the SAM file non-uniquely aligned reads were discarded. Replicates of control and treated

samples were pooled into two data sets "Control" and "Treated". Then, junction coordinates were determined by applying an in-house developed pipeline, which incorporates SAMTOOLS and parses the CIGAR read alignment string. Read alignments containing insertions and/or deletions or large clippings were discarded. As a result, for each junction the numbers of supporting reads were determined for both the GSNAP and STAR alignment. Junctions were discarded if they were detected in both treated and control samples, as well as represented in the Intropolis database and/or in RefSeq/Ensembl annotations. We use only junctions that are supported by at least one read count in the treated samples and no read count in the control samples by both aligners GSNAP and STAR. The majority (80.7%) of those junctions are not in RefSeq/Ensembl annotations. (see Supplementary Table 3). However, more than half of the junctions are represented in the Intropolis database by both (55.4%) or one (39.3%) of the two junction coordinates. Only 38 junctions (5.3%) detected in the treated but not in the control samples were not observed elsewhere.

Gene Set Enrichment Analysis (GSEA). The edgeR[28] algorithm was used for differential gene expression analysis and identified genes with absolute log2 fold change larger than 1 and Benjamini–Hochberg adjusted p value smaller than 0.05 as significantly changed. The camera[29] algorithm was applied for gene set enrichment analysis with gene sets collected in the Roche internal database RONET. The same method was applied for upstream regulator analysis, with transcriptional targets of human transcription factors and gene expression modulators curated from literature in RONET as input. Gene ontology enrichment analysis was performed using the Fisher's exact test.

Motifs analysis. For the motif search 41 cassette exons were selected that show the strongest splicing effects measured in ΔPSI. For the background distribution 1'051 cassette exons were selected that show weak or no splicing effects but are alternatively spliced. They were selected based on ΔPSI values less than 0.1 and PSI values between 0.1 and 0.9. From these cassette exons we analyzed 40 nucleotide long sub-sequences surrounding the acceptor and donor sites: For the acceptor site we collected sub-sequences including 20 nucleotides upstream of the exon and the first 20 nucleotides of the exon and for the donor site the last 20 nucleotides of the exons

and the first 20 nucleotides downstream of the exon. The four motif-finding algorithms Gibbs[30] version 3.10.00, MEME[31] version 4.10.0, Weeder[32] version 2.0, and Homer[33] version 4.7.2 were applied using these sequence sets. WebLogo[34] version 2.8.2 was used to generate sequence logos. All input scripts used are available upon request.

Molecular docking

For the RNA preparation, the 40 conformations of the NMR bundle were clustered with the XCluster tool of Maestro 10.0 (Schrödinger suite of programs, www.schrodinger.com) based on RMSD. One representative structure per cluster was chosen and prepared using the Protein Preparation Wizard of Maestro 10.0. Bond orders were assigned and H-bonds optimized using a pH of 6.4, matching the NMR experimental conditions. An all-atom restrained minimization was carried out by the maximum RMSD of 0.3 Å in order to avoid steric clashes, and structures were saved as mae and pdb files. During ligand preparation, PK4C9 and its structural analogues were first drawn from scratch, and 3D conformations were obtained using the LigPrep tool of Maestro 10.0. The OPLS_2005 force field was chosen and all the possible ionization states were created at a pH of 6.4. The Glide 6.5 program (Schrödinger) was used for molecular docking. A receptor grid box (15 Å) was built and centred between base pairs A1-U19 and U2-G18. The default settings were maintained and the standard precision mode of GlideScore was selected as the scoring function. After docking calculation, poses were energy minimized.

Molecular dynamics

A total of 12 MD trajectories of 40-ns or 100-ns with explicit solvent were produced using the AMBER12 suite of programs (University of California, San Francisco)[35]. The starting points of the RNA in pentaloop and triloop conformations were obtained from the NMR structures, whereas the RNA-PK4C9 complexes were extracted from the docking solutions. The Biopolymer toolkit of Sybyl x2.1.1 (www.certara.com) was used to generate the mutant RNA structures. These mutated models underwent a first minimization step (steepest descent method) in order to remove potential steric clashes introduced by the replacement of the base. All the systems were parameterized using the AMBER ff12SB force field (which includes the refined parmbsc0 and parmχOL modifications for

RNA[36,37]) with the AMBER program Leap. The ligand charge was assigned from the general AMBER force field (GAFF) using the Antechamber and Parmchk programs[38]. The systems were solvated with water TIP3P into an octahedral box of 12Å. A net-neutralized sodium charge was then applied to each system with a supplementary 50 mM of NaCl in order to mimic the NMR experimental conditions. All the structures then underwent the same minimization, heating and equilibration protocols: I) a first 5000-step minimisation step, where restrains were applied to nucleic acids and ligand (steepest descendent and conjugated gradient methods were applied), followed by a second 5000-step minimization where the entire system was allowed to move. II) During the heating phase the temperature increases from 0 K to 298 K using the Langevin thermostat, and with 100 kcal mol^{-1} $Å^{-2}$ positional restrains for RNA, ligand and ions over the course of the first 80 ps at constant pressure (1 atm). The restrain forces were gradually turned off to zero in the following 100 ps, allowing movement of all atoms. III) A final heating and equilibrating step was performed at 300 K at constant pressure over 820 ps. Both for the heating and equilibration steps, a collision frequency of 1.0 ps^{-1} was applied to control Langevin dynamics. Finally the MD production was carried out for 40 ns per system using the NPT ensemble (constant pressure, 1 atm; and temperature, 300 K). The simulation lengths, of the ligand-free triloop and pentaloop TSL2 trajectories, as well as TSL2pentPK4C9, were extended to 100 ns to confirm the events sampled within 40 ns. Coordinates were recorded every picosecond. The SHAKE algorithm was used to restrain the lengths of all bonds involving hydrogen atoms, allowing an increase of the time step up to 2 fs with a cut-off space of 9 Å. The default particle-mesh Ewald settings (which correspond to a grid spacing of ~1 Å and a direct space tolerance of 10^{-6}) were used to determine long-range charge interactions. All the MD production simulations were performed using the PMEMD program of AMBER12, whereas Sander (AMBER12) was used during minimization, heating and equilibration. CPPTRAJ of AmberTools[39] was used for processing and analysis of MD trajectories. Representative MD structures were obtained using the average-linkage clustering algorithm. All structure images were visualized and processed using the VMD software. All input scripts used are available upon request.

Chemical library construction and compound synthesis

A focused collection of 304 small molecules with favoured RNA-binding scaffolds (indole, benzimidazole, alkyl pyridinium and 2-phenyl benzimidazole) was built in-house using ligand-based modeling; taken from a larger, highly-diverse in-house library of >3000 compounds. In-house synthesis of PK4C9 (CAS Registry Number: 172286-77-0) and its derivatives BJGF466 (CAS Registry Number: 630110-69-9), 001 (CAS Registry Number: 474816-40-5), 002 (CAS Registry Number: 630110-77-9), 003 (CAS Registry Number: 847448-70-8), 004 (CAS Registry Number: 630110-68-8), 005 (CAS Registry Number: 847448-72-0), and 007 (deoxytopsentin, CAS Registry Number: 112515-42-1) were carried out according to the literature[40-45]. ^{1}H, ^{13}C NMR and MS data of target compounds were comparable to those reported in the literature[40-45]. PK4C9 analogues 006 and 008 (pyridine-2,6-diylbis((1*H*-indol-3-yl)methanone) were new compounds, described below.

Analogue 006: Melting-point: amorphous

^{1}H-NMR (300 MHz, $CDCl_3$): *d* = 1.22 (t, 3H, *J* = 7.0 Hz, CH_3), 3.66 (q, 2H, *J* = 7.0 Hz, OCH_2Me), 5.99 (s, 2H, NCH_2), 7.21-7.48 (m, 6H, H_{indole}), 7.61 (s, 1H, $H_{imidazole}$), 7.62 (d, 1H, *J* = 3.0 Hz, H_{indole}), 8.04-8.07 (m, 1H, H_{indole}), 8.29 (br. s, 1H, NH), 8.55 (d, 1H, *J* = 7.7 Hz, H_{indole}), 8.94 (br. s, 1H, NH), 9.01 (d, 1H, *J* = 3.1 Hz, H_{indole}) ppm.

^{13}C-NMR (75 MHz, $CDCl_3$): *d* = 15.0 (CH_3), 65.2 (CH_2), 77.2 (CH_2), 109.6 (C), 112.0 (2 x CH), 116.0 (C), 118.4 (CH), 119.7 (CH), 120.4 (CH), 122.1 (CH), 122.5 (CH), 122.9 (CH), 123.1 (CH), 123.7 (CH), 124.9 (C), 126.6 (C), 136.1 (C), 136.4 (C), 136.5 (C), 137.4 (CH), 143.2 (C), 178.1 (CO) ppm.

HRMS (ESI): calcd. For $C_{23}H_{21}N_4O_2$ $[M + H]^+$ 385.1659, found 385.1648.

Analogue 008: Melting-point: > 210 °C

^{1}H-NMR (300 MHz, DMSO-d_6): *d* = 7.25-7.32 (m, 4H, H_{indole}), 7.49-7.55 (m, 2H, H_{indole}), 8.24-8.27 (m, 3H, $H_{pyridine}$), 8.41-8.46 (m, 2H, H_{indole}), 8.69 (d, 2H, *J* = 2.9 Hz, H_{indole}), 12.07 (br. s, 2H, NH) ppm.

^{13}C-NMR (100 MHz, DMSO-d_6): *d* = 112.3 (2 x CH), 113.7 (2 x C), 121.8 (2 x CH), 122.3 (2 x CH), 123.3 (2 x CH), 125.0 (2 x CH), 126.9 (2 x C), 136.2 (2 x C), 138.0 (2 x CH), 139.0 (CH), 154.9 (2 x C), 185.8 (2 x CO).

HRMS (ESI): calcd. For $C_{23}H_{15}N_3NaO_2$ $[M + Na]^+$ 388.1056, found 388.1046.

Finally, NCI377363 was obtained from the National Cancer Institute repository (NCI Plated 2007). 288D was previously described[46].

Circular dichroism

Non-labelled TSL2 RNAs (10 μM) were snap annealed in 25 mM Tris-HCl pH 7.5, 100 mM NaCl, 5 mM $MgCl_2$ by heating to 90 °C for 5 min and placing on ice. RNA spectra between 320 and 200 nm were obtained at 10 °C (unless otherwise stated) in 0.5 mm cuvettes with a J-815 spectropolarimeter (Jasco). 10 measurements per collection point (data pitch 0.2 nm) were averaged.

2-amino purine (2AP) assay

2AP was introduced at position 15 of synthetic TSL2 RNA, substituting its analogue G. 2AP-labelled RNA (1 μM) was snap annealed in 8 mM Na_2HPO_4 pH 7, 185 mM NaCl, 0.1 mM EDTA, 40 μg per mL BSA, and incubated with DMSO (control) or ligand (100 μM) for 30 min in black 96-well plates (quadruplicates). Fluorescence emission was measured between 340 and 440 nm in a Synergy™ Mx (BioTek) plate reader (excitation at 312 nm). Emissions of buffer or ligands alone were subtracted from the final RNA spectra. 2AP fluorescence changes were calculated by dividing emission at the peak (365 nm) of RNA with ligand by the emission at the peak of the RNA alone.

Native PAGE

Non-labelled TSL2 RNAs (10, 30 and 100 μM) were snap annealed in 25 mM Tris-HCl pH 7.5, 100 mM NaCl, 5 mM $MgCl_2$, and loaded into prerun (15', 80V) 16 % polyacrylamide non-denaturing gels. Electrophoresis was carried out per triplicate at 4 °C in TBE 0.5X for ~90 min at 150 V, followed by 20 min staining with Sybr Safe (Invitrogen) and visualization under UV light. Quantification of band intensity was performed on ImageJ. Denaturing PAGE using Urea gels (8M) were run in parallel as a control.

Cytotoxicity

Cells were seeded per sextuplicate at a density of 1 x10^4 cells per well in 96-well plates (200 μL per well) and cultivated for 24 h at 37 °C in 5% CO_2. Each plate was repeated twice, giving a total of 12

replicates per point. Cells were then treated for 24 h or 72 h and the Cytotoxicity Detection kit (LDH) from Roche was used to measure cell death in a Synergy™ Mx (BioTek) plate reader at 492 nm and 600 nm (reference) according to the manufacturer's instructions. DMSO-treated cells were used as the baseline (i.e., 0 % death reference) and chemically lysed cells were used as the 100 % death reference.

Differential Scanning Fluorimetry (DSF)

DSF with RliboGreen (300 nM) was used to calculate the melting temperatures of n.m. TSL2 and mutant TSL2 RNAs (600 nM) in 96 well plates, as previously described[47] (n=6).

Supplementary figures

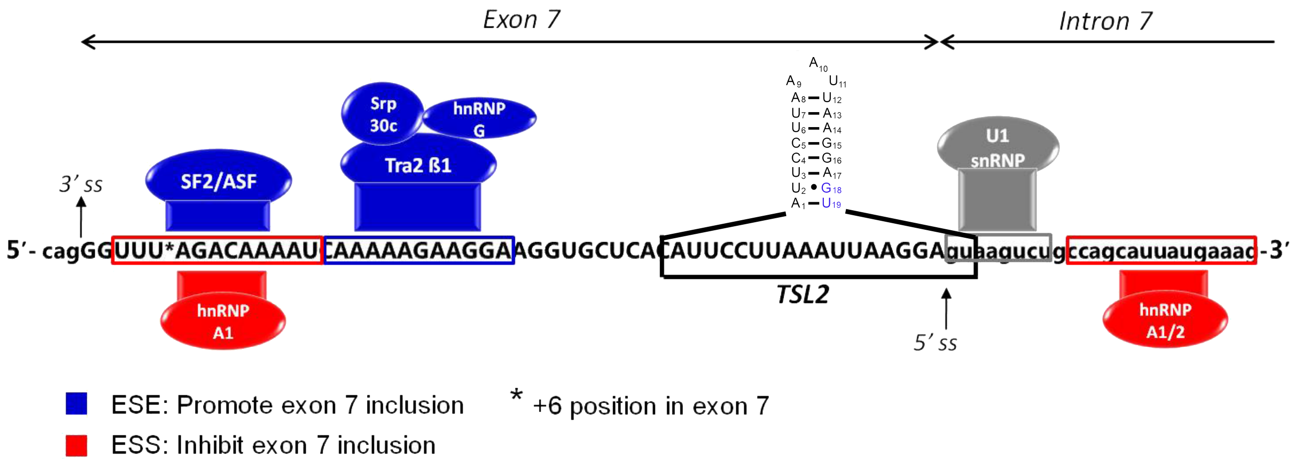

Supplementary Figure 1. TSL2 location at the exon 7/intron 7 junction of the *SMN* transcripts. Diagram of exon 7 (E7; capital letters) and adjacent intronic sequence (small case) of the *SMN* transcripts. Trans and cis-elements, which promote (blue) and inhibit (red) E7 inclusion, are indicated. TSL2 (Terminal Stem-Loop 2) and its partial overlap with the 5 ’ss are outlined. The predicted secondary structure of TSL2 is depicted. This figure was generated by the authors.

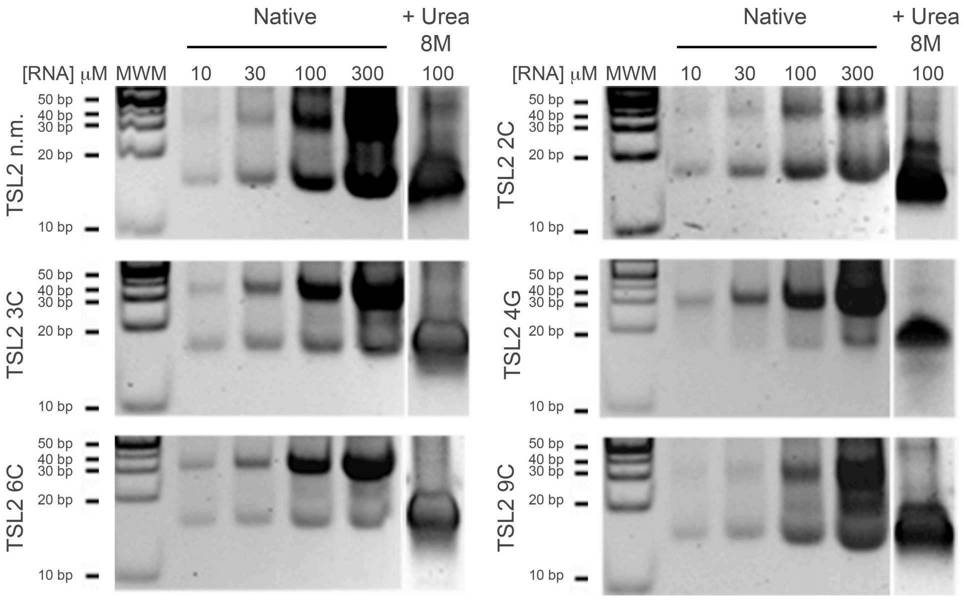

Supplementary Figure 2. Visualization of *in vitro* TSL2 folding using polyacrylamide gel electrophoresis (PAGE). Native PAGE of synthetic, non-mutated (n.m.) TSL2 or synthetic TSL2 RNAs with the indicated mutations, folded by snap cooling (n=3). In all cases, the presence of TSL2 hairpin was detected (lower band), together with a higher molecular weigh band, the size of which is consistent with an RNA duplex. This upper band increased with RNA concentration (10, 30, 100 and 300 µM), supporting its duplex nature. Denaturing electrophoresis using Urea (8M) revealed only one band, confirming that the two bands detected under native conditions represent folding states and not contaminants from the RNA synthesis. MWM, molecular weight marker.

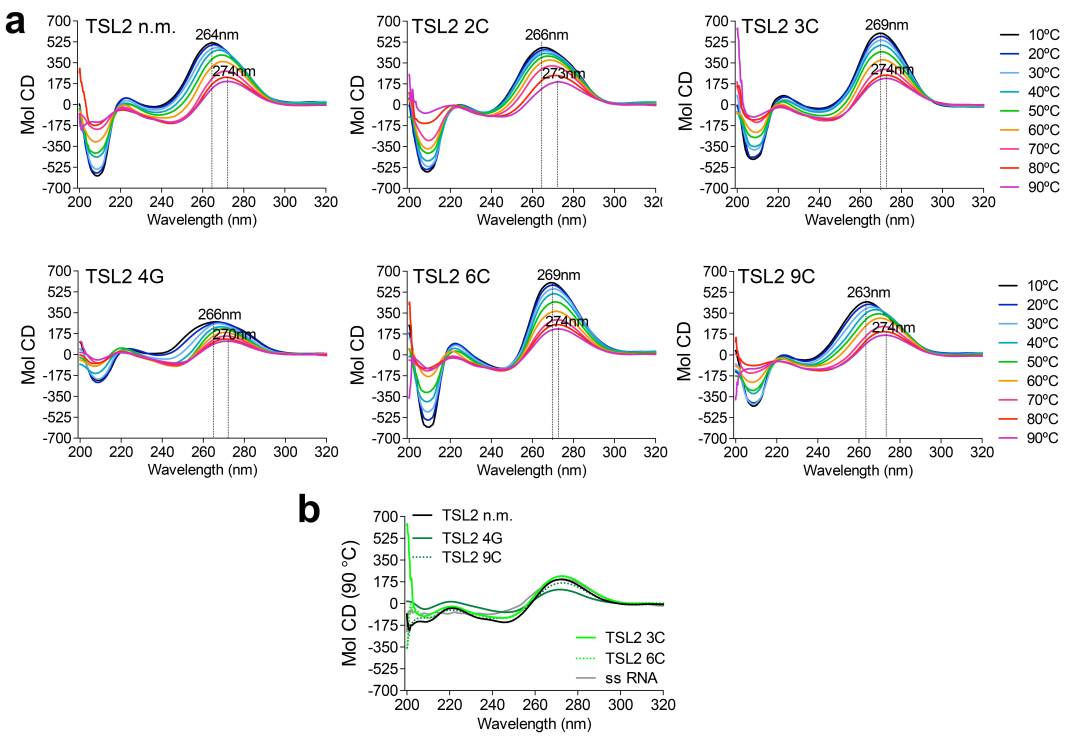

Supplementary Figure 3. Circular Dichroism (CD) thermal spectra scanning of TSL2. (a) Temperature scan (10 ˚C to 90 ˚C) of synthetic, non-labeled TSL2 RNAs in its non-mutated form (TSL2 n.m.) or carrying the indicated mutations. The positive peak of all mutated forms differ from the n.m. TSL2 at temperatures where RNA is structured, but not at 90 ˚C (melted RNA, b) (n=10 scans). Compare with Fig. 1f. Positive CD peaks at ~265 nm are typical of RNA. A negative peak at ~210 nm is typical of the A-form. Reduced intensity of the negative peak indicates loss of helicity. A reduction in the positive CD signal indicates loss of base stacking. Wavelength shifts suggest additional rearrangements. ss RNA, single stranded RNA (control). Mol CD, Molar Circular Dichroism units.

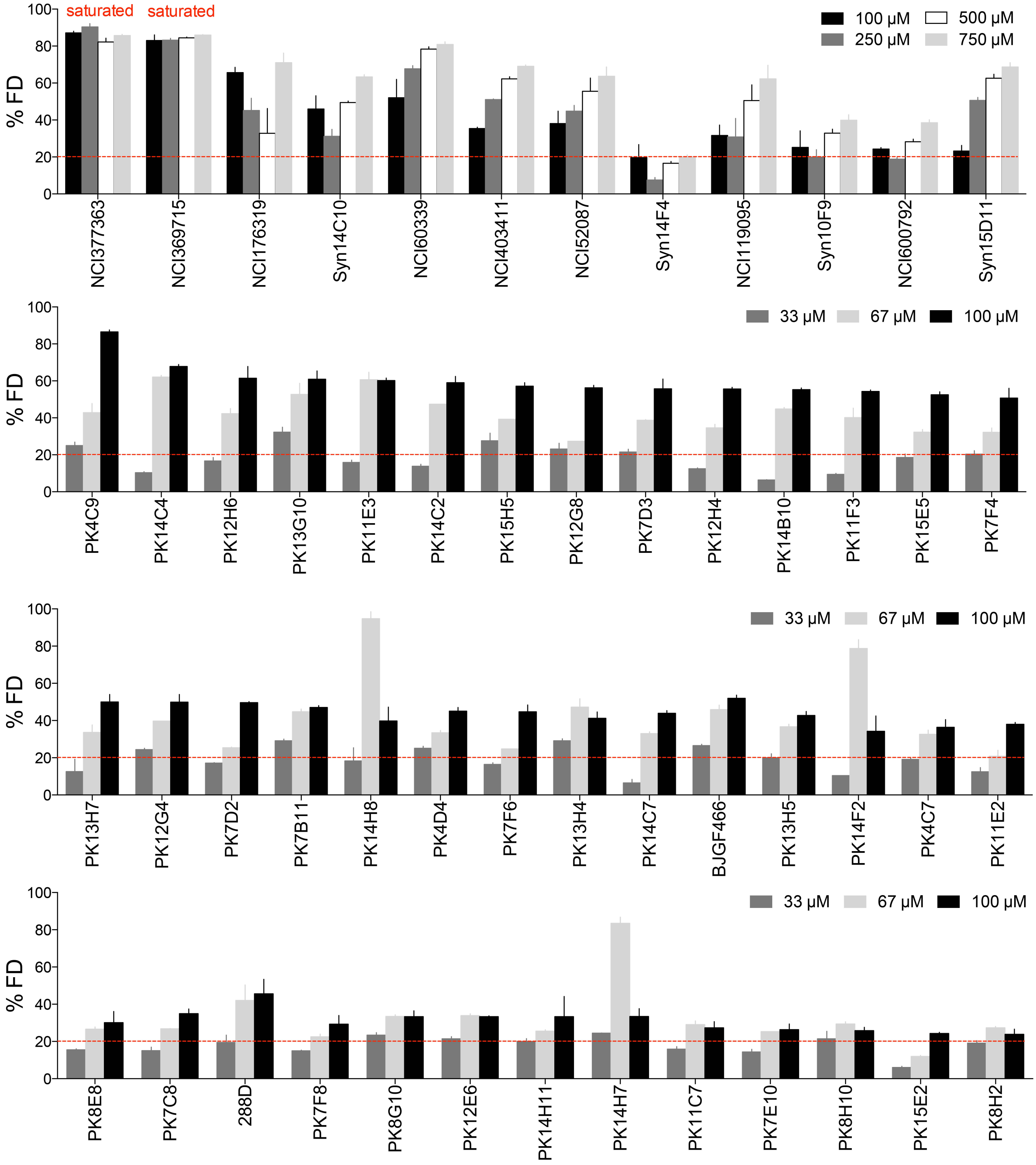

Supplementary Figure 4. Dose-response of primary TSL2-interacting hits. 45 out of the 54 hits from the primary fluorescence displacement (FD) screening showed good dose-response behaviour (n=4). All graphs represent mean values ± SEM.

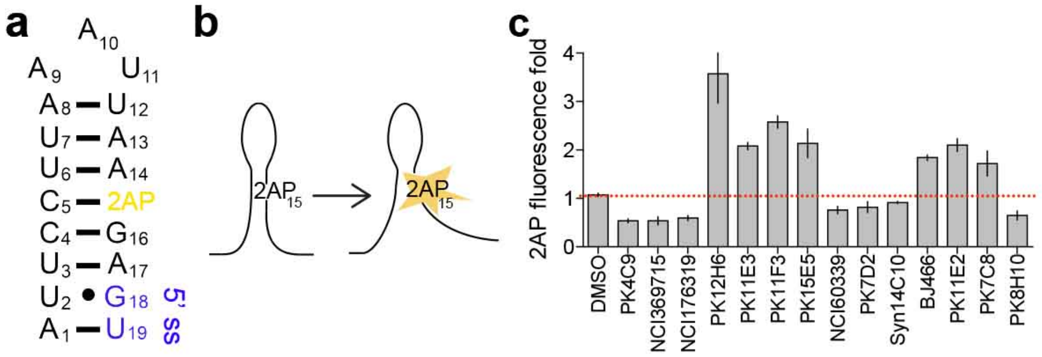

Supplementary Figure 5. Primary TSL2-interacting hits with a conformational effect on TSL2. (a) Diagram of the synthetic 2-amino purine (2AP)-labelled TSL2 RNA. 2AP was introduced at position 15, substituting its analogue G. (b) The fluorescence of 2AP is strongly quenched in a base-paired environment, but it increases as the harboring RNA structure relaxes. (c) 14 out of 28 hits from the primary FD screening modified TSL2 base stacking, as measured by changes in the fluorescence of 2AP at the peak of maximum emission (365 nm) compared to the control wells (1% DMSO) (n=4). The remaining 17 hits from the 45 primary hits with good dose-response could not be tested due to autofluorescence interfering with the emission of 2AP. Graph represents mean values ± SEM.

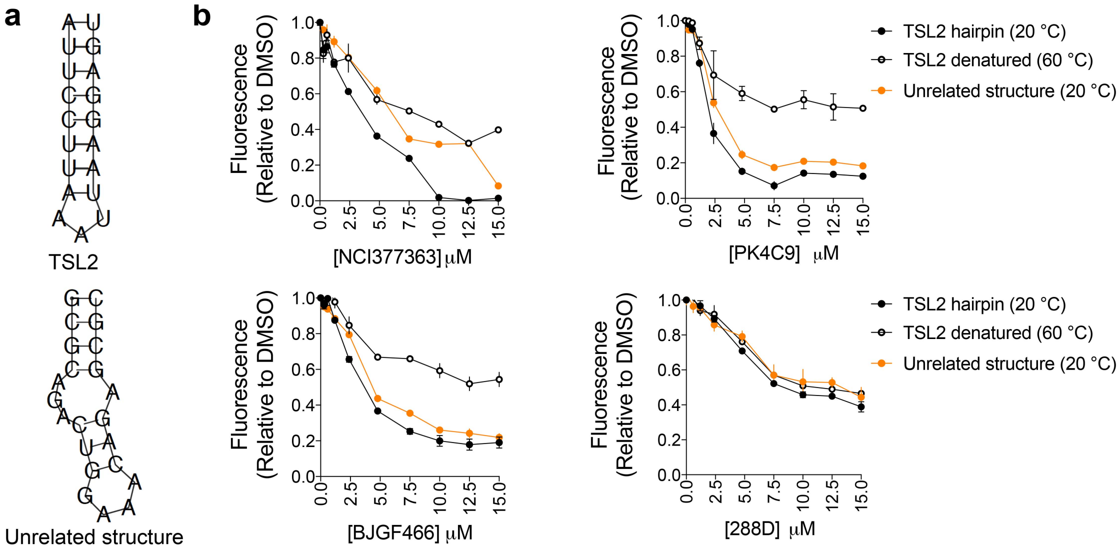

Supplementary Figure 6. Fluorescence displacement assay for binding selectivity. (a) A fluorescence displacement assay was performed to rule out pan-nucleic acid binding, using the RiboGreen dye. Binding of our most promising hits (NCI377363, PK4C9, BJG466, and 288D) to native TSL2 was compared with binding to the following controls: an unrelated RNA structure and a partially denatured TSL2 (60 °C). The sequence and predicted secondary structures (RNAfold online tool) of both RNAs are shown. (b) NCI377363, PK4C9, and BJG466 showed significantly higher fluorescence displacement with native TSL2 than with the control unrelated RNA structure, indicating some binding selectivity. 288D showed weak binding to both. A dramatic decrease in NCI377363, PK4C9 and BJGF466 binding was observed when TSL2 was, at least partially, unfolded (Tm of TSL2 under the assay conditions is 45.6 °C), indicating that these hits target TSL2 structure rather than its primary sequence, and that they do not efficiently bind to single-strand RNA. Conversely, 288D binding was not affected. Collectively, these results demonstrate that NCI377363, PK4C9 and BJGF466 are not promiscuous RNA binders, whereas 288D seems to be. Data points represent mean values ± SEM (n=6). RiboGreen can bind to double stranded and to single stranded nucleic acids.

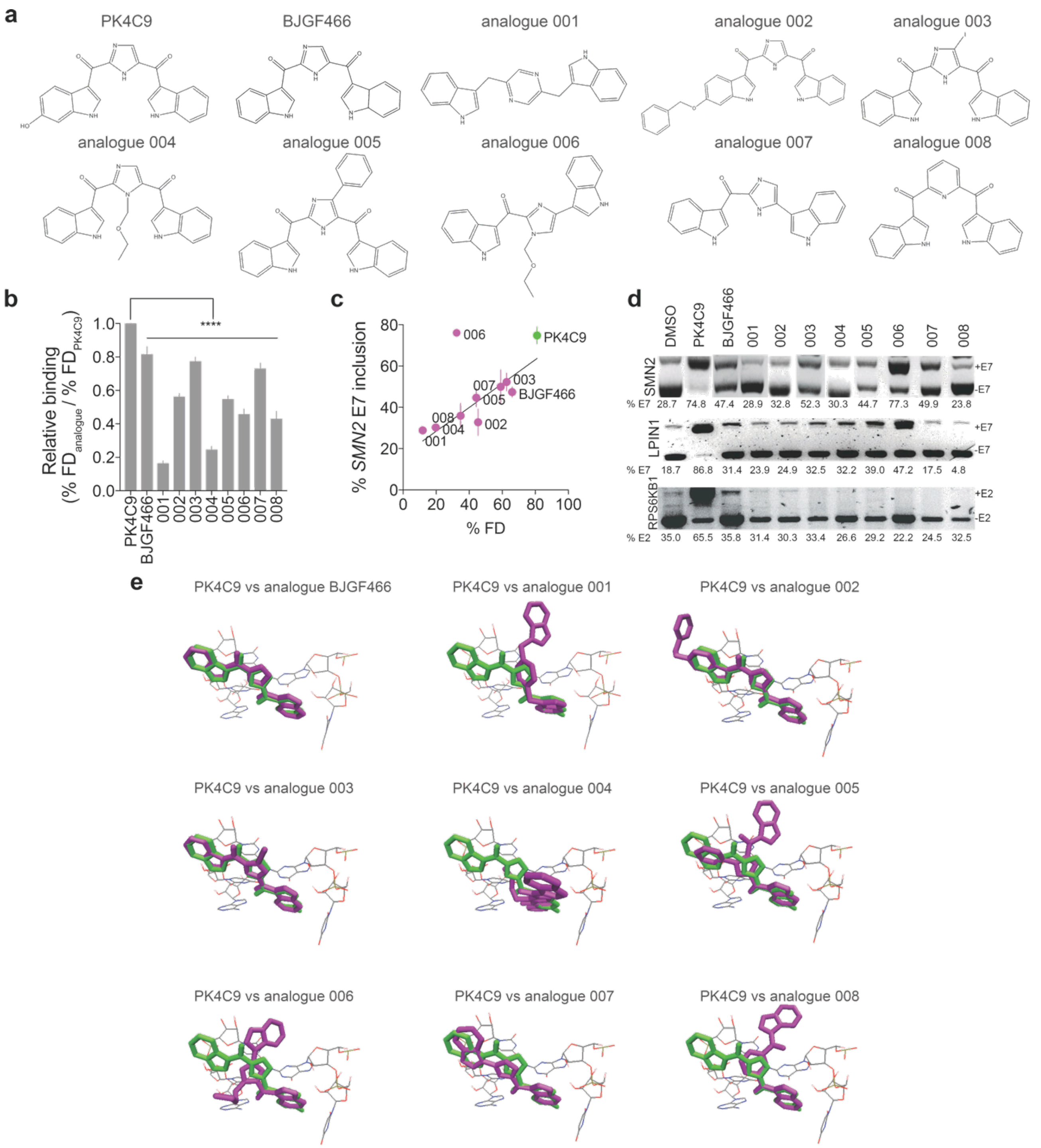

Supplementary Figure 7. Structural analogues of PK4C9. (a) Chemical structures of PK4C9, BJGF466 and additional PK4C9 analogues (001-to-008) that were designed and synthesized in house. (b) All structural analogues of PK4C9 showed reduced binding to TSL2 by fluorescence displacement (FD, n=4). Binding values are shown normalized to PK4C9. (c) PK4C9 analogues also had reduced *SMN2* E7 splicing modifier activity in transfected HeLa cells (40 μM, 24 h; n=3), which significantly correlated with their TSL2-binding levels (R^2 0.90), with the exception of

analogue 006. This exception could be explained by the compensatory effect of other parameters (f.e., membrane permeability; logP 006 = 3.3 *vs.* logP PK4C9 = 1.5). 006 was excluded from the curve fitting. (d) Agarose gel showing RT-PCR bands of *SMN2* E7 splicing, as well as two PK4C9-sensitive splicing events that depend on SMN levels: *LPIN1* E7 and *RPS6KB1* E2 (also see Fig. 4d). RT-PCR was performed on SMA fibroblasts treated with PK4C9, BJGF466 and analogues 001-to-008 at 40 μM or 0.04 % DMSO (control) for 24 h (n=3 biological replicates). (e) Predicted binding pose of PK4C9 analogues to TSL2 as per molecular docking. No binding pose could be identified for analogues 001 and 004 that recapitulated the pose of PK4C9. Consistently, these two analogues displayed the lowest binding to TSL2 and the lowest *SMN2* splicing modifier activity (b, c). **** $p<0.0001$. Graph shows mean values ± SEM. p-values were obtained by applying non-paired, two-tailed *t* tests with Bonferroni and Welch corrections.

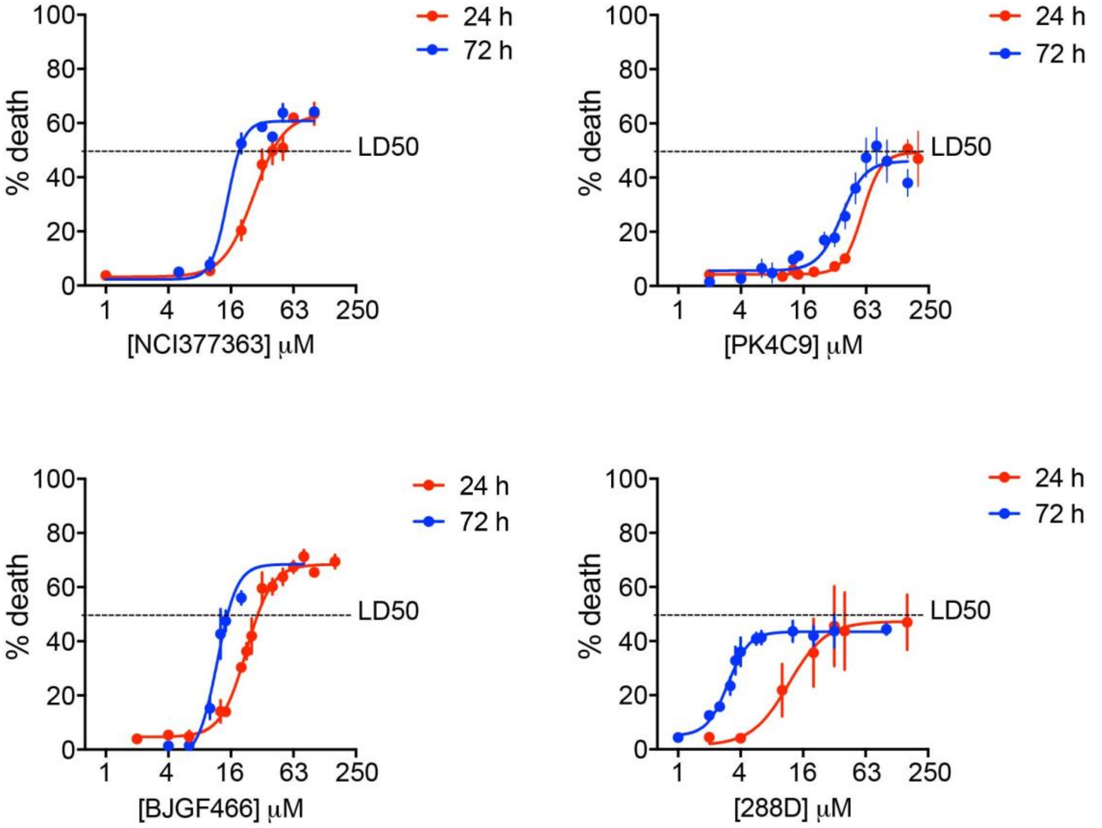

Supplementary Figure 8. Cytotoxicity of splicing modifier hits in GM03814B fibroblasts. To determine the dose window between the activity and toxicity of our splicing modifier hits, we obtained cell death curves after 24 h and 72 h of treatment in GM03814B fibroblasts, using the lactate dehydrogenase (LDH) assay (n=12; 6 biological replicates and 2 technical replicates). The percentage of death of compound-treated cells was calculated relative to DMSO-treated cells. A cell lysis agent was used as the 100 % death control. LD50 were not calculated, since none of the molecules reached 100 % cell death at any concentration. Of the six hits, PK4C9 showed the lowest percentage of cell death (also see Main Fig. 3d). Data points represent mean values ± SEM.

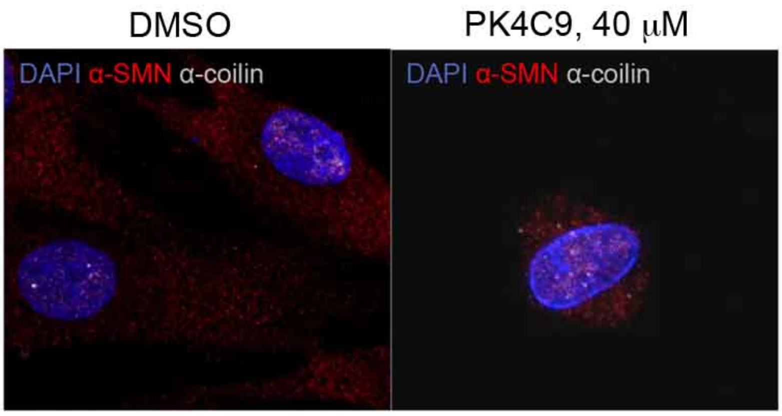

Supplementary Figure 9. PK4C9 restores SMN in GM03813C cells. Double antibody staining showing the increase in SMN (red) in the cytoplasm and nucleus of GM03813C fibroblasts after treatment with PK4C9 (40 μM), and its restored localization in nuclear gems (coilin-p80, white; quantified in Fig. 4c). (n=30 cells from 3 biological replicates).

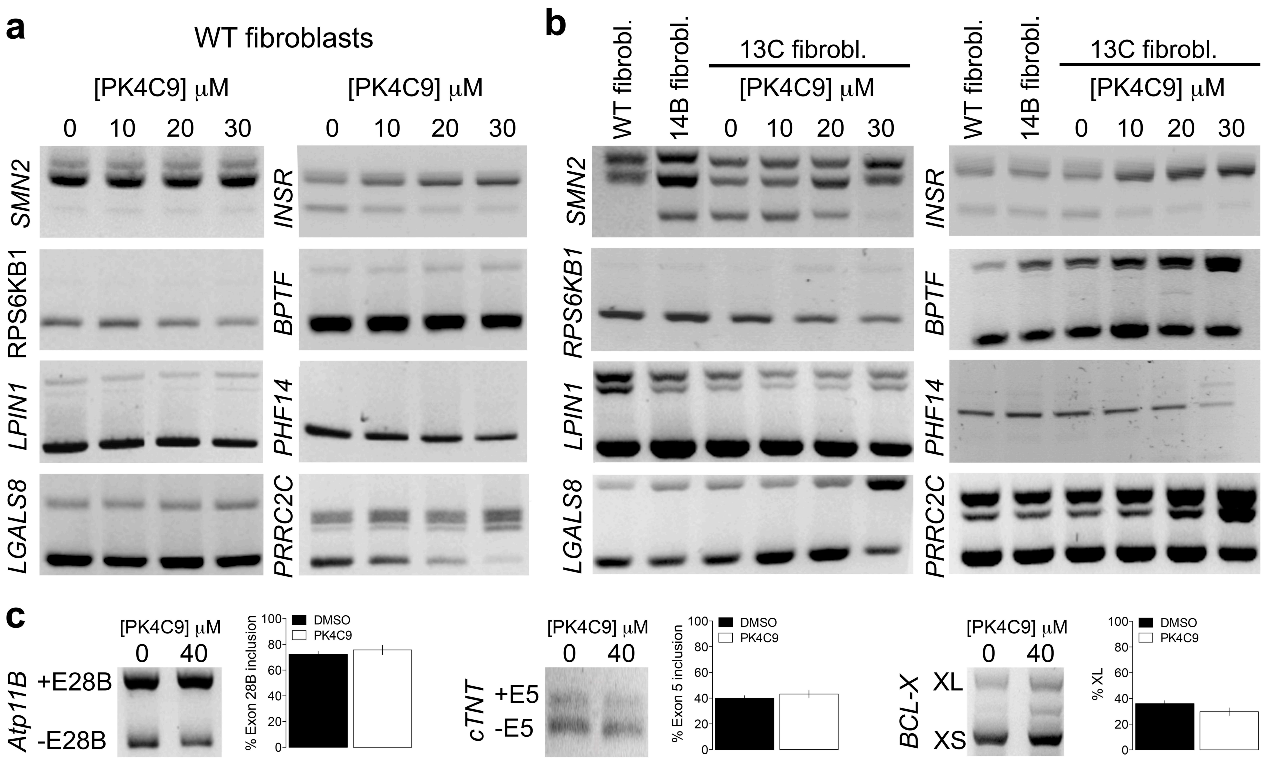

Supplementary Figure 10. Effect of PK4C9 on the splicing of SMN-regulated transcripts. Agarose gels from RT-PCR of ND36091A (WT, a), GM03814B (b) and GM03813C (SMA, b) fibroblasts showing the effect of PK4C9 (24 h) on selected transcripts known to be SMN-regulated[48-50] and identified as PK4C9-senstive in our RNA-seq experiment (see also Fig. 4d). *SMN2* is shown as a positive control. (c) RT-PCR of transcripts the splicing of which is not SMN-mediated and that were not affected in our RNA-seq experiment, used here as negative controls. All bars represent mean values ± SEM. (n=8; 4 biological replicates and 2 technical replicates)

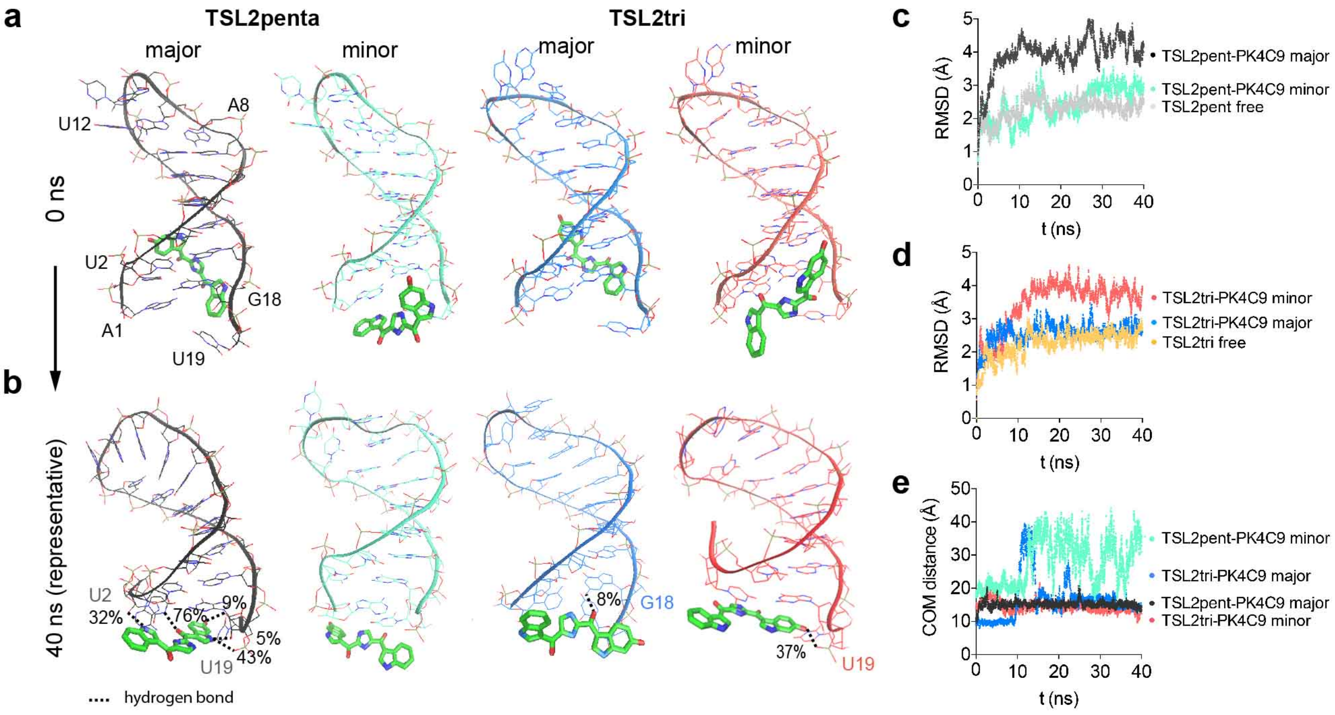

Supplementary Figure 11. Generation of the starting PK4C9 binding model to TSL2. One representative triloop conformer (TSL2tri) and one pentaloop conformer (TSL2penta) were selected from the 40 NMR structures as the unbound state of TSL2 (see Fig. 6). (a) Ligand docking was applied to both structures in order to build a starting 3D model of the binding mode of PK4C9 to the terminal region of TSL2, using a 15 Å grid around residues A1 and U2, and G18 and U19. Two starting binding sites per structure were found, one in the major groove and one in the minor groove. (b) To obtain a dynamic view of the evolution of these binding predictions, and assess their stability, 40-ns MD simulations were conducted for each model, using the structures in (a) as starting coordinates. The TSL2penta-PK4C9 major complex (hereafter referred to as TSL2pent-PK4C9) showed the most stable interaction, with a reorganization of the ligand binding orientation that allowed the highest frequency of hydrogen bonds with residues U2 and U19 (see also Supplementary Movies 1 and 2). This stable complex was chosen for all subsequent structure-activity analysis, and its MD trajectory was extended to 100 ns. Percentages (%) indicate hydrogen bond frequency. (c, d) The RMSD of all simulations, which measures the deviation from the stating coordinates of all structures generated during the MD trajectory, reached equilibrium in all the studied cases, confirming the stability of our NMR structures and of our modeled systems. (e)

Analysis of the distance between the center of mass (COM) of PK4C9 and the COM of TSL2 supports that the TSL2pent-PK4C9 major model is the most stable.

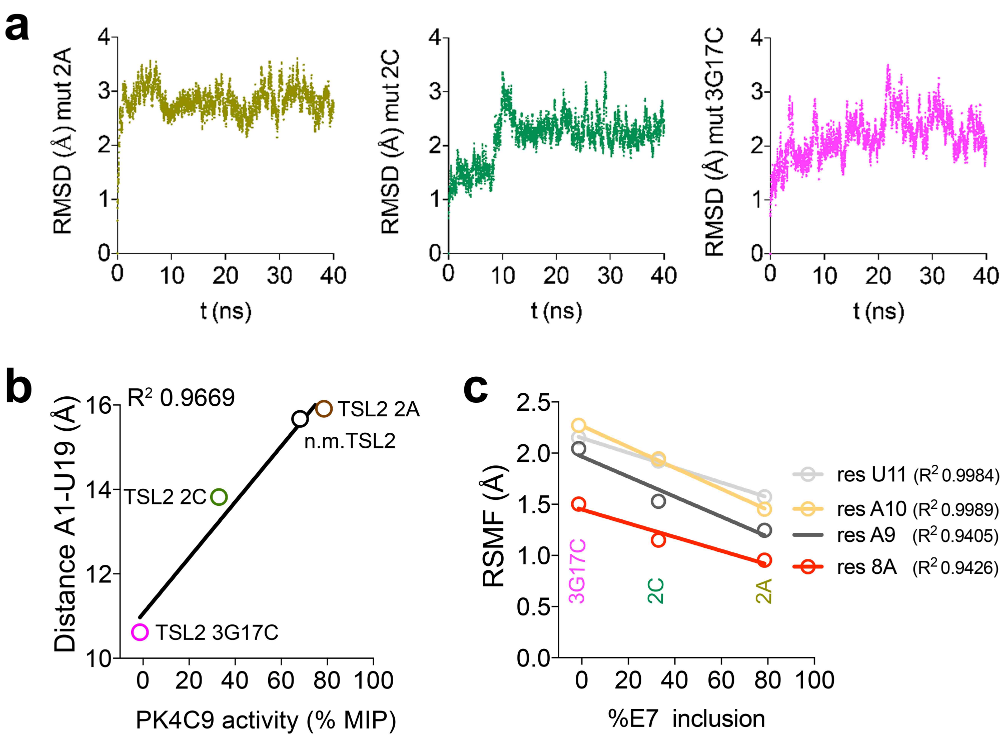

Supplementary Figure 12. RMSD of TSL2 mutants and structural correlations with E7 splicing. (a) The NMR structure of TSL2 in its pentaloop conformation was mutated *in silico* to generate TSL2 mutants 2A, 2C, and 3G17C. These mutant versions of TSL2 were used as starting points in 40-ns MD simulations. RMSD values confirmed that all three trajectories reached equilibrium early in the simulation. (b) Terminal TSL2 opening at the 5' ss in n.m., 2A, 2C, and 3G17C correlates with PK4C9 activity. MIP, maximum E7 increment possible (see equation 2). (c) The mobility of loop residues A8-to-U11 inversely correlates with PK4C9 activity.

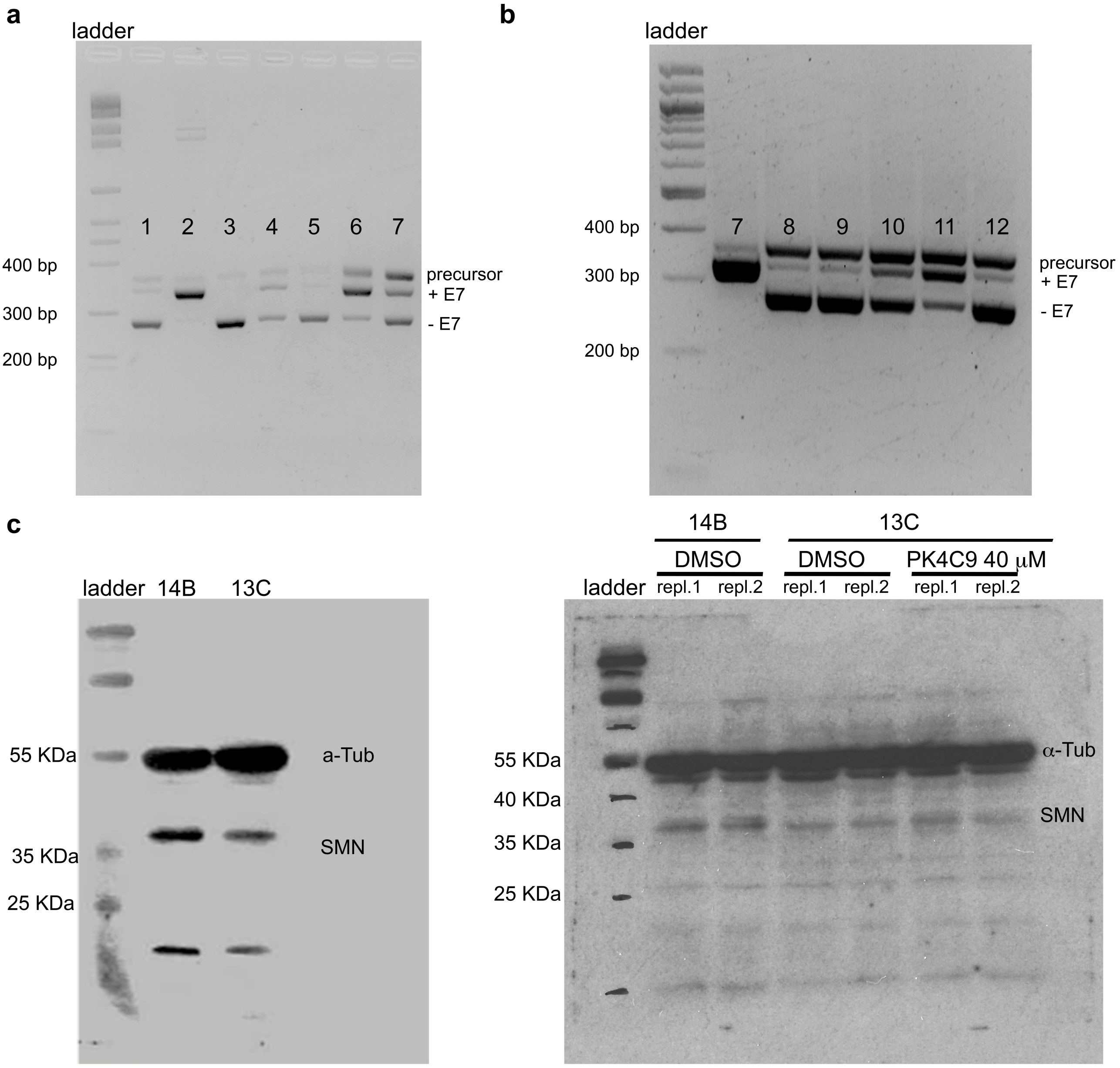

Supplementary Figure 13. Full agarose gel and western blot images. (a) Full RT-PCR agarose gel image of Fig. 1b. 1, *SMN2*; 2, *SMN1*; 3, *SMN1* (TSL2 2C); 4, *SMN2* (TSL2 3C); 5, *SMN2* (TSL2 4G); 6, *SMN2* (TSL2 6C); 7, *SMN2* (TSL2 9C). (b) Full RT-PCR agarose gel image of Fig. 3a (PK4C9, 24 h). 7, *SMN1*; 8, *SMN2* + DMSO; 9, *SMN2* + PK4C9 10 μM; 10, *SMN2* + PK4C9 20 μM; 11, *SMN2* + PK4C9 40 μM; 12, *SMN2* + PK4C9 80 μM. In (a, b) the higher molecular weigh band corresponds to precursor *SMN* mRNA[51]. (c) Full western blot scan image of Fig 4a.

Supplementary tables

Supplementary Table 1. Summary of results for structural mutations in TSL2

TSL2 mutant	% E7 (HeLa)	2AP	% hairpin	CD effect (*vs.* WT)	∆PK4C9 activity	Tm	MD
**WT**	16.7 ± 2.8	37.9± 0.6	34	-	-	45.6 ± 0.1	triloop & pentaloop
**2A**	39.18 ±2.4	n.d.	n.d.	n.d.	18.4	51.5 ± 0.1	↑ triloop
**2C**	10.9 ± 1.2	80.9 ± 0.6	53	n.d.	-27.2	51.8 ± 0.1	triloop & pentaloop
**2G**	67.9 ± 1.6	n.d.	n.d.	n.d.	-6.8	42.0 ± 0.0	n.d.
**3A**	42.1 ±3.8	n.d.	n.d.	n.d.	10.0	51.1 ± 0.1	n.d.
**3C**	54.7 ± 5.2	145.4 ± 5.7	19	peak shift	-19.6	44.7 ± 0.1	n.d.
**3G**	68.4 ± 3.1	n.d.	n.d.	n.d.	8.3	49.2 ± 0.1	n.d.
**4G**	21.5 ± 1.5	307.8 ±11.9	8	↓ signal	-6.8	48.7 ± 0.1	n.d.
**6C**	62.6 ± 4.5	205.5 ± 3.9	13	peak shift	-16.9	41.2 ± 0.2	n.d.
**9C**	47.6 ± 6.7	90.3 ± 0.3	28	↓ signal	-10.4	44.7 ± 0.1	n.d.
**17C**	86.3 ± 0.8	n.d.	n.d.	n.d.	12.2	47.6 ± 0.0	n.d.
**8G12C**	95.5 ± 1.0	n.d.	n.d.	n.d.	n.d.	52.2 ± 0.0	n.d.
**3G17C**	12.5 ± 2.7	n.d.	n.d.	n.d.	-61.5	51.0 ± 0.1	↑ pentaloop

% E7, percentage of E7 inclusion (Fig.1c); 2AP, 2-amino purine (Fig. 1d); % hairpin, in native PAGE (Fig. 1e); CD, Circular Dichroism (Fig. 1f); Tm, melting temperature obtained by differential scanning fluorimetry (DSF) as described in[47]; MD, Molecular Dynamics (Fig. 7); ΔPK4C9 activity, increment in PK4C9 activity. All values represent mean ± SEM, except for % hairpin and ∆PK4C9 activity, which were calculated pooling all replicates together.

Supplementary Table 2. TSL2-binding hits tested for E7 splicing

			HeLa (1)			GM03813C (2)		
	**Molecule**	**[ ] μM**	**E7 inclusion fold (mean ± SEM)**	**p-value**	**signify-cance**	**E7 exclusion fold (mean ± SEM)**	**p-value**	**signify-cance**
**1**	PK4C9	40	3.8 ± 0.3	< 0.0001	****	0.06 ± 0.01	< 0.0001	****
**2**	BJGF466	40	2.8 ± 0.1	< 0.0001	****	0.7 ± 0.2	0.0102	*
**3**	288D	40	2.6 ± 0.1	< 0.0001	****	0.7 ± 0.08	0.0003	***
**4**	NCI377363	10	2.5 ± 0.3	< 0.0001	****	0.3 ± 0.09	< 0.0001	****
**5**	PK7C8	40	2.3 ± 1.7	0.0369	*	not tested		
**6**	PK12G8 -Cl	40	2.1 ± 0.2	0.0003	***	0.7 ± 0.3	0.0377	*
**7**	Syn14F4	40	1.9 ± 0.1	0.0002	***	0.9 ± 0.04	0.0364	*
**8**	NCI176319	20	1.7 ± 0.1	0.0156	*	not tested		
**9**	PK12H6	2	1.4 ± 0.1	0.1645	n.s.			
**10**	PK11F3	40	1.2 ± 0.3	0.4678	n.s.			
**11**	PK12G8	40	1.3 ± 0.3	0.5056	n.s.			
**12**	PK7D2	35	1.2 ± 0.1	0.3647	n.s.			
**13**	PK15E5	35	1.1 ± 0.3	0.6377	n.s.			
**14**	PK14C4	40	1.1 ± 0.03	0.0562	n.s.			
**15**	PK11E2	40	1.0 ± 0.1	0.9301	n.s.			
**16**	PK11E3	40	0.97 ± 0.3	0.9311	n.s.			
**17**	Syn14C10	35	0.7 ± 0.1	0.3534	n.s.			
**18**	PK12E6	40	0.3					
**19**	NCI369715	10	0.2					

(1) RT-PCR of exons 6-to-8 from HeLa cells transfected with *SMN*$^{E6\text{-}7\text{-}8}$ minigenes and treated for 24 h at the indicated concentrations. (2) SMA fibroblasts, qRT-PCR of endogenous exon 7 exclusion after 24 h of treatment at 40 μM (PK4C9, BJGF466, 288D, PK12G8-Cl, Syn14F4) or 20 μM (NCI377363) (n=4). NCI176319 and PK7C8 were not tested on GM03813B cells due to toxicity. * p<0.05, *** p<0.001, **** p<0.0001. n.s, non-significant. p-values were obtained by applying non-paired, two-tailed *t* tests with Bonferroni and Welch corrections.

Supplementary Table 3. Numbers of junctions represented in Intropolis and RefSeq/Ensembl GTF exon annotation

	Intropolis Database	RefSeq/Ensembl GTF
begin and end coordinates present	399 (55.4%)	3 (0.4%)
only begin coordinate present	138 (19.2%)	62 (8.6%)
only end coordinate present	145 (20.1%)	74 (10.3%)
begin and end coordinate not present	38 (5.3%)	581 (80.7%)
**Sum**	**720**	**720**

Supplementary Table 4. RNA and DNA oligos

RNA oligos

Oligo	Label	Sequence	Used in
WT TSL2	none	AUUCCUUAAAUUAAGGAGU	CD, PAGE, FD, NMR
39C TSL2	none	ACUCCUUAAAUUAAGGAGU	CD, PAGE
40C TSL2	none	AUCCCUUAAAUUAAGGAGU	CD, PAGE
41G TSL2	none	AUUGCUUAAAUUAAGGAGU	CD, PAGE
43C TSL2	none	AUUCCCUAAAUUAAGGAGU	CD, PAGE
46C TSL2	none	AUUCCUUACAUUAAGGAGU	CD, PAGE
54G TSL2	none	AUUCCUUAAAUUAAGGGGU	CD, PAGE
ssRNA	none	CAUUCCUUAAACCGUCCGAAA	CD
2AP-WT TSL2	2-amino purine	AUUCCUUAAAUUAAG[2AMP]GAGU	fluorim.
2AP-39C TSL2	2-amino purine	ACUCCUUAAAUUAAG[2AMP]GAGU	fluorim.
2AP-40C TSL2	2-amino purine	AUCCCUUAAAUUAAG[2AMP]GAGU	fluorim.
2AP-41G TSL2	2-amino purine	AUUGCUUAAAUUAAG[2AMP]GAGU	fluorim.
2AP-43C TSL2	2-amino purine	AUUCCCUAAAUUAAG[2AMP]GAGU	fluorim.
2AP-46C TSL2	2-amino purine	AUUCCUUACAUUAAG[2AMP]GAGU	fluorim.
2AP-54G TSL2	2-amino purine	AUUCCUUAAAUUAAG[2AMP]GGGU	fluorim.
^{13}C-^{15}N-A-TSL2	^{13}C10, ^{15}N5 in all adenines	AUUCCUUAAAUUAAGGAGU	NMR

DNA oligos for semi-quantitative PCR

Oligo	Sequence (5'→3')	Tm, cycles
SMN2 and SMN1 minigenes, HeLa		
P1 (fw)	CGACTCACTATAGGCTAGCC	58°C, 30x
P2 (rev)	GCATGCAAGCTTCCTTTTTTCTTTCCCAACAC	58°C, 30x
Endongenous SMN2, fibroblasts		
N24 (fw)	CCAGATTCTCTTGATGATGCTGATGCTTTGGG	58°C, 30x
P2 (rev)	GCATGCAAGCTTCCTTTTTTCTTTCCCAACAC	58°C, 30x
SMN2 minigene, Drosophila		
SMN2 (fw)	TCTGATGCTTTGGGAGTATGTT	60°C, 30x
SMN2 (rev)	CAGCGTAAGTGATGTCCACCT	60°C, 30x
Others		
cTNTf	ATAGAAGAGGTGGTGGAAGAGTAC	58°C, 30x
cTNTr	GTCTCAGCCTCTGCTTCAGCATCC	58°C, 30x
Atp11Bf	GCTCCGGATCGATCCTGAGAACT	58°C, 30x
Atp11Br	GTAACCATTATAAGCTGCAA	58°C, 30x
INSRf	CCAAAGACAGACTCTCAGAT	58°C, 30x
INSRr	AACATCGCCAAGGGACCTGC	58°C, 30x
BCL-Xf	GGAGCTGGTGGTTGACTTTCT	58°C, 30x
BCL-Xr	TAGAAGGCACAGTCGAGG	58°C, 30x
RPS6KB1f	ACGCTGAACTTTAGGAGCCA	58°C, 30x
RPS6KB1r	AGTTCATATGGTCCAACTCCCC	58°C, 30x
BPTFf	CCCCAACAAGCAGTACAACC	58°C, 30x
BPTFr	AGGTTTGCTGACTGGTACCT	58°C, 30x
LPIN1f	AGGAAAACCTCTCCCTGGC	58°C, 30x
LPIN1r	GCTCTCCCCACAGCCAAA	58°C, 30x
PHF14f	AGAGTTCCTAGAGAGAGAAGACA	58°C, 35x
PHF14r	ACATATCCATCCGTAGCCTGT	58°C, 35x
LGALS8f	ACCCAAGCATCTAGTCTGGA	58°C, 30x
LGALS8r	CGTGGGTTCAAGTGTAGAGC	58°C, 30x
PRRC2Cf	GAACAGCCAGTCCAGCAAAA	58°C, 30x
PRRC2Cr	GCCTCCTCCTATCTGTTTGC	58°C, 30x
CPSF6f	CGTTTTCCAGGGGCTGTTC	58°C, 30x
CPSF6r	GGAGGAAGTGGACCCAATG	58°C, 30x

DNA oligos and Taqman probes for qPCR

Oligo	[nM]	Sequence (5'→3')
SMN2 FL	900	GCTCACATTCCTTAAATTAAGGAGAAA
SMN2 ΔE7	900	TGGCTATCATACTGGCTATTATATGGAA
SMN2rev	900	TCCAGATCTGTCTGATCGTTTCTT
Taqman SMN2	250	6FAM-CTGGCATAGAGCAGCACTAAATGACACCAC- TAMRA
GAPDHf	400	CAACGGATTTGGTCGTATTGG

GAPDHr	400	TGATGGCAACAATATCCACTTTACC
Taqman GAPDH	125	VIC-GCCTGGTCACCAGGGCTGCT–TAMRA
INSR-FL_fw	100	CCCCAGAAAAACCTCTTCAGG
INSR_rev	100	GTCACATTCCCAACATCGCC
Rps6kb1_FL_fw	100	TCTTCCAATCTTCCAGGGGTC
Rps6kb1_rev	100	TTCATATGGTCCAACTCCCCC
Lpin1ΔE7fw	100	TCACCCACTCCCAGTCCTTC
Lpin1_rev	100	CCTTTCCGTGGACTTGCTGA
PHF14_FL_fw	100	TCTTGTCAGCAGGATGAGATTGT
PHF14_rev	100	ACATATCCATCCGTAGCCTGT
Prrc2c_FL_fw	100	GTCCTAGGTGTTGGCAAAGC
Prrc2c_rev	100	TCAGAGAAGAACCGTTGCGT

All RNA oligos were synthetized by Microsynth AG, GE Dharmacon, Thermo Fisher Scientific or Sigma Aldrich.

All DNA oligos and probes were synthetized by Microsynth AG or Thermo Fisher Scientific.

Supplementary References

1. Sklenář, V. & Bax, A. Spin-echo water suppression for the generation of pure-phase two-dimensional NMR spectra. *J Magn Res* **74**, 469-479 (1987).
2. Schanda, P., Kupče, Ē. & Brutscher, B. SOFAST-HMQC Experiments for Recording Two-dimensional Deteronuclear Correlation Spectra of Proteins within a Few Seconds. *J Biomol NMR* **33**, 199-211 (2005).
3. Jeener, J., Meier, B.H., Bachmann, P. & Ernst, R.R. Investigation of exchange processes by two-dimensional NMR spectroscopy. *J Chem Phys* **71**, 4546-4546 (1979).
4. Wagner, R. & Berger, S. Gradient-Selected NOESY—A Fourfold Reduction of the Measurement Time for the NOESY Experiment. *J Magn Res A* **123**, 119-121 (1996).
5. Bax, A. & Davis, D.G. MLEV-17-based two-dimensional homonuclear magnetization transfer spectroscopy. *J MagRes* **65**, 355-360 (1985).
6. Bodenhausen, G. & Ruben, D.J. Natural abundance nitrogen-15 NMR by enhanced heteronuclear spectroscopy. *Chem Phys Lett* **69**, 185-189 (1980).
7. Breeze, A.L. Isotope-filtered NMR methods for the study of biomolecular structure and interactions. *Prog Nucl Magn Reson Spectrosc* **36**, 323-372 (2000).
8. Ogura, K., Terasawa, H. & Inagaki, F. An improved double-tuned and isotope-filtered pulse scheme based on a pulsed field gradient and a wide-band inversion shaped pulse. *J Biomol NMR* **8**, 492-498 (1996).
9. Iwahara, J., Wojciak, J.M. & Clubb, R.T. Improved NMR spectra of a protein-DNA complex through rational mutagenesis and the application of a sensitivity optimized isotope-filtered NOESY experiment. *J Biomol NMR* **19**, 231-241 (2001).
10. Davis, A.L., Keeler, J., Laue, E.D. & Moskau, D. Experiments for recording pure-absorption heteronuclear correlation spectra using pulsed field gradients. *J Magn Res* **98**, 207-216 (1992).
11. Kay, L.E., Xu, G.Y., Singer, A.U., Muhandiram, D.R. & Formankay, J.D. A gradient-enhanced HCCH-TOCSY experiment for recording side-chain 1H and 13C correlations in H2O samples of proteins. *J Magn Res B* **101**, 333-337 (1993).
12. Palmer, A.G., Cavanagh, J., Wright, P.E. & Rance, M. Sensitivity improvement in proton-detected two-dimensional heteronuclear correlation NMR spectroscopy. *J Magn Res* **93**, 151-170 (1991).
13. Vuister, G.W. & Bax, A. Resolution enhancement and spectral editing of uniformly ^{13}C-enriched proteins by homonuclear broadband ^{13}C decoupling. *J Mag Res* **98**, 428-435 (1992).
14. Ebrahimi, M., Rossi, P., Rogers, C. & Harbison, G.S. Dependence of ^{13}C NMR Chemical Shifts on Conformations of RNA Nucleosides and Nucleotides. *J Magn Rese* **150**, 1-9 (2001).
15. Ohlenschläger, O., Haumann, S., Ramachandran, R. & Görlach, M. Conformational signatures of 13C chemical shifts in RNA ribose. *J Biomol NMR* **42**, 139-142 (2008).
16. Brünger, A.T., *et al.* Crystallography & NMR system: A new software suite for macromolecular structure determination. *Acta Crystallograph. D Biol Crystallogr* **54**, 905-921 (1998).
17. Linge, J.P., O'Donoghue, S.I. & Nilges, M. Automated assignment of ambiguous nuclear overhauser effects with ARIA. *Methods Enzymol* **339**, 71-90 (2001).
18. Nozinovic, S., Fürtig, B., Jonker, H.R.A., Richter, C. & Schwalbe, H. High-resolution NMR structure of an RNA model system: the 14-mer cUUCGg tetraloop hairpin RNA. *Nucleic Acids Res* **38**, 683-694 (2010).
19. Kneller, D.G. & Goddard, T.D. SPARKY. (University of California, San Francisco).

20. Linge, J.P., Williams, M.A., Spronk, C.A., Bonvin, A.M. & Nilges, M. Refinement of protein structures in explicit solvent. *Proteins* **50**, 496-506 (2003).
21. Hwang, T.L. & Shaka, A.J. Water suppression That works. Excitation sculpting using arbitrary wave-forms and pulsed-field gradients. *J Magn Res A* **112**, 275-279 (1995).
22. Schleucher, J., *et al.* A general enhancement scheme in heteronuclear multidimensional NMR employing pulsed field gradients. *J Biomol NMR* **4, 301-306**(1994).
23. Kay, L., Keifer, P. & Saarinen, T. Pure absorption gradient enhanced heteronuclear single quantum correlation spectroscopy with improved sensitivity. *J Am Chem Soc* **114**, 10663-10665 (1992).
24. Wu, T.D. & Watanabe, C.K. GMAP: a genomic mapping and alignment program for mRNA and EST sequences. *Bioinformatics* **21**, 1859-1875 (2005).
25. Wu, T.D. & Nacu, S. Fast and SNP-tolerant detection of complex variants and splicing in short reads. *Bioinformatics* **26**, 873-881 (2010).
26. Mortazavi, A., Williams, B.A., McCue, K., Schaeffer, L. & Wold, B. Mapping and quantifying mammalian transcriptomes by RNA-Seq. *Nat Methods* **5**, 621-628 (2008).
27. Li, H., *et al.* The sequence alignment/map format and SAMtools. *Bioinformatics* **25**, 2078-2079 (2009).
28. Robinson, M.D., McCarthy, D.J. & Smyth, G.K. edgeR: a Bioconductor package for differential expression analysis of digital gene expression data. *Bioinformatics* **26**, 139-140 (2010).
29. Wu, D. & Smyth, G.K. Camera: a competitive gene set test accounting for inter-gene correlation. *Nucleic Acids Res* **40**, e133 (2012).
30. Lawrence, C.E., *et al.* Detecting subtle sequence signals: a Gibbs sampling strategy for multiple alignment. *Science* **262**, 208-214 (1993).
31. Bailey, T.L. & Elkan, C. Fitting a mixture model by expectation maximization to discover motifs in biopolymers. *Proc Int Conf Intell Syst Mol Biol* **2**, 28-36 (1994).
32. Pavesi, G., Mereghetti, P., Mauri, G. & Pesole, G. Weeder Web: discovery of transcription factor binding sites in a set of sequences from co-regulated genes. *Nucleic Acids Res* **32**, W199-203 (2004).
33. Heinz, S., *et al.* Simple combinations of lineage-determining transcription factors prime cis-regulatory elements required for macrophage and B cell identities. *Mol Cell* **38**, 576-589 (2010).
34. Crooks, G.E., Hon, G., Chandonia, J.M. & Brenner, S.E. WebLogo: a sequence logo generator. *Genome Res* **14**, 1188-1190 (2004).
35. Case, D.A., Darden, T.A., Cheatham, III, T.E., Simmerling, C.L., Wang, J., Duke, R.E., Luo, R., Walker, R.C., Zhang, W., Merz, K.M., Roberts, B., Hayik, S., Roitberg, A., Seabra, G., Swails, J., Götz, A.W., Kolossváry, I., Wong, K.F., Paesani, F., Vanicek, J., Wolf, R.M., Liu, J., Wu, X., Brozell, S.R., Steinbrecher, T., Gohlke, H., Cai, Q., Ye, X., Wang, J., Hsieh, M.-J., Cui, G., Roe, D.R., Kovalenko, A. & Kollman P.A. AMBER 12. *University of California, San Francisco* (2012).
36. Perez, A., *et al.* Refinement of the AMBER force field for nucleic acids: improving the description of alpha/gamma conformers. *Biophys J* **92**, 3817-3829 (2007).
37. Zgarbova, M., *et al.* Refinement of the Cornell *et al.* Nucleic acids force field based on reference quantum chemical calculations of glycosidic torsion profiles. *J Chem Theory Comput* **7**, 2886-2902 (2011).
38. Wang, J., Wolf, R.M., Caldwell, J.W., Kollman, P.A. & Case, D.A. Development and testing of a general amber force field. *J Comput Chem* **25**, 1157-1174 (2004).

39. Roe, D.R. & Cheatham, T.E., 3rd. PTRAJ and CPPTRAJ: Software for processing and analysis of molecular dynamics trajectory data. *J Chem Theory Comput* **9**, 3084-3095 (2013).
40. Montagne, C., Fournet, G. & Joseph, B. Synthesis of homocarbonyltopsentine derivatives. *Synthesis*, 136-146 (2005).
41. Montagne, C., Fournet, G. & Joseph, B. An efficient synthetic route to homocarbonyltopsentine. *Synlett*, 1533-1535 (2003).
42. Shaaban, M., Maskey, R.P., Wagner-Dobler, I. & Laatsch, H. Pharacine, a natural p-cyclophane and other indole derivatives from Cytophaga sp strain AM13.1. *J Nat Prod* **65**, 1660-1663 (2002).
43. Bartik, K., *et al.* Topsentins, New Toxic bis-indole alkaloids from the marine sponge topsentia-genitrix. *Can J Chem* **65**, 2118-2121 (1987).
44. Tsujii, S., *et al.* Topsentin, bromotopsentin, and dihydrodeoxybromotopsentin - antiviral and antitumor bis(indolyl)imidazoles from Caribbean deep-sea sponges of the family halichondriidae - structural and synthetic studies. *J Org Chem* **53**, 5446-5453 (1988).
45. Veale, C.G., *et al.* Synthetic analogues of the marine bisindole deoxytopsentin: potent selective inhibitors of MRSA pyruvate kinase. *J Nat Prod* **78**, 355-362 (2015).
46. Weinreb, S.M., Garigipati, R.S. & Gainor, J.A. Natural product synthesis via syclo-additions with N-sulfinyl dienophiles. *Heterocycles* **21**, 309-324 (1984).
47. Silvers, R., Keller, H., Schwalbe, H. & Hengesbach, M. Differential scanning fluorimetry for monitoring RNA stability. *Chembiochem* **16**, 1109-1114 (2015).
48. Zhang, Z., *et al.* Dysregulation of synaptogenesis genes antecedes motor neuron pathology in spinal muscular atrophy. *Proc Natl Acad Sci U S A* **110**, 19348-19353 (2013).
49. Zhang, Z., *et al.* SMN deficiency causes tissue-specific perturbations in the repertoire of snRNAs and widespread defects in splicing. *Cell* **133**, 585-600 (2008).
50. Baumer, D., *et al.* Alternative splicing events are a late feature of pathology in a mouse model of spinal muscular atrophy. *PLoS Genet* **5**, e1000773 (2009).
51. Singh, N.N., Singh, R.N. & Androphy, E.J. Modulating role of RNA structure in alternative splicing of a critical exon in the spinal muscular atrophy genes. *Nucleic Acids Res* **35**, 371-389 (2007).
