# Supplementary material for: Targeting RNA structure in SMN2 reverses spinal muscular atrophy molecular phenotypes
Source: Nat Commun. 2018 May 23;9:2032. doi: 10.1038/s41467-018-04110-1 (PMC5966403; doi:10.1038/s41467-018-04110-1)
Supplement: Supplementary file 3 — Description of Additional Supplementary Files [file 41467_2018_4110_MOESM3_ESM.docx]

**Description of Additional Supplementary Files**

File Name: Supplementary Data 1

Description: Primary results from the TSL2-binding screening by FD

File Name: Supplementary Data 2

Description: Numbers of junctions represented in Intropolis and RefSeq/Ensembl GTF exon annotation

File Name: Supplementary Data 3

Description: Differentially spliced transcripts from the RNA-seq analysis selected for validation by qPCR

File Name: Supplementary Movie 1

Description: Static 3D view of the binding mode of PK4C9 to TSL2, taken from the representative cluster structure of a 100-ns MD trajectory.

File Name: Supplementary Movie 2

Description: Dynamic 3D view of the binding mode of PK4C9 to TSL2 throughout a 100-ns MD trajectory.
